# HIV/AIDS, Media, and Health Journalism: Exploring Empathetic Journalism Practices in HIV/AIDS Reporting on the Mass Media

**DOI:** 10.12688/f1000research.187439.1

**Published:** 2026-08-07

**Authors:** Mas'amah Mas'amah, Monika Wutun, Herman Elfridus Seran, Apris A. Adu, Emanuel Sowe Leuape

**Affiliations:** 1 Communication Science, University of Nusa Cendana, Kupang, East Nusa Tenggara, Indonesia; 2 Communication Science, University of Nusa Cendana, Kupang, East Nusa Tenggara, Indonesia; 3 Communication Science, University of Nusa Cendana, Kupang, East Nusa Tenggara, Indonesia; 4 Public Health Science, University of Nusa Cendana, Kupang, East Nusa Tenggara, Indonesia; 5 Communication Science, University of Nusa Cendana, Kupang, East Nusa Tenggara, Indonesia

**Keywords:** HIV/AIDS, Health Journalism, Mass Media, Empathy, Framing Pan & Kosicki

## Abstract

**Background:**

This study explores the practices of health journalism in mass media coverage of Human Immunodeficiency Virus and Acquired Immunodeficiency Syndrome (HIV/AIDS) during World HIV/AIDS Day on the online media platform Pos-Kupang.com. It evaluates whether online news is written from a perspective of empathy towards people living with HIV (PLHIV) or simply centers on tracking case statistics and reporting negative case distributions.

**Methods:**

The research employed Zhongdan Pan and Gerald M. Kosicki’s Framing Analysis model, operationalizing four structural text dimensions as framing devices: Syntactic, Script, Thematic, and Rhetorical. Data collection was conducted virtually by selecting all active news stories related to HIV/AIDS published by Pos-Kupang.com during December 2023.

**Results:**

Out of eight dedicated news stories monitored, only two were deemed to have been written about the active human struggles of PLHIV to overcome social stigma and discrimination. The overall framing output was dictated by daily media routines and individual journalist characteristics, including their professional backgrounds, personalities, and personal attitudes.

**Conclusions:**

Local online mass media platforms establish routine reporting columns for World AIDS Month but fail to incorporate empathetic journalism values. Media reporting structures reduce human suffering into statistical objects, reinforcing the urgent need for structured health journalism training to foster supportive public health communication.

## Introduction

The increase in HIV and AIDS cases is a worrying phenomenon. This is evidenced by the increasing number of cases each year. It is important to note that humans have lived side by side with HIV and AIDS since it was first discovered globally in 1920 and in Indonesia in 1987 ( Andina, 2019). Data on the development of the increase in cases in Indonesia has increased significantly from year to year. At the time of this study, data from the Indonesian Ministry of Health (Kemenkes) recorded that the number of HIV cases in Indonesia was projected to reach 515,455 cases during January–September 2023 ( Hasibuan et al., 2024). Of this total, 454,723 cases, or 88%, had been confirmed by sufferers or people living with HIV (PLHIV). The Ministry of Health stated that only 40% of people living with HIV (PLHIV) are receiving HIV treatment. By age, the majority of HIV sufferers in Indonesia are in the 25–49 age group, accounting for 69.9% of the total cases. The second largest age group is the 20–24 age group, at 16.1%. This is followed by those aged 50 and over at 7.7% and those aged 15–19 at 3.4%. Children, including toddlers or those aged 4 and over, account for 1.9% and those aged 5–14 at 1% ( Pratiwi et al., 2022). The distribution of HIV/AIDS cases can also be found in Kupang City, which shows a quite alarming number of cases in recent years.

In the context of HIV/AIDS, mass media plays a strategic role as a channel for disseminating information related to HIV/AIDS, quickly reaching the wider community ( Indriani & Zainuddin, 2025). Through various content produced, mass media can convey messages related to education, prevention, and management of HIV/AIDS. Functionally, mass media plays a crucial role in health communication, encompassing education, monitoring, and instilling values in the community through the content produced ( Solihin et al., 2023). However, in reality, there are still media reports that prioritize the increase in cases over reports written from the perspective of sufferers, so that the information consumed by the public ultimately contributes to the formation of a negative stigma against PLWHA ( Yani et al., 2020). This concern started from the decrease in the amount of budget support for handling HIV and AIDS in Kupang City and NTT (source: Primary Data of the Kupang City AIDS Commission (KPA), mass media that very rarely report on HIV and AIDS cases each year even from the results of virtual observations conducted by researchers found that mass media in NTT, especially cyber media, very rarely report on HIV and AIDS. If there is news related to HIV and AIDS, the media only focuses on bombastic topics such as increasing numbers of cases, risky behavioral deviations that can have an impact on HIV/AIDS ( Wutun, 2018).

The reality obtained by researchers even on World AIDS Day on December 1st each year, there are only a few media that pay attention by broadcasting news about HIV and AIDS. And from the results of these observations, it was found that local mass media categorized as cyber media that paid attention to the issue of HIV and AIDS by broadcasting this news twice on World AIDS Day was Pos-kupang.com which can be accessed at https://kupang.tribunnews.com/. Meanwhile, other cyber mass media in Kupang City only reported once or did not report topics related to HIV and AIDS at all. The search was carried out again by researchers to find news about HIV and AIDS in mass media in NTT which have editorial headquarters in Kupang City and it turned out the harsh reality was found. Not all media treat HIV and AIDS as a common problem. In a period of one year (12 months) alone, the frequency of news on the same topic by mass media in the province and Kupang City was very limited.

This limited number of news items raises the question of whether all news published by the mass media in Kupang City is “friendly” to HIV and AIDS. The term “friendly” refers to the bias in reporting towards people living with HIV and AIDS (PLWHA) or people living with HIV and AIDS (PLWHA), as well as volunteers and humanitarians who dedicate their lives to preventing and addressing the negative impacts of this disease, which has become a global epidemic. In the realm of journalism, reporting that supports PLWHA, PLWHA, and the humanitarian struggle related to HIV and AIDS can be approached with the concept of empathetic journalism, which can be implemented by the mass media in Kupang City, including being understood and implemented by journalists. Empathetic journalism is used in news with high human interest value, such as news about HIV and AIDS, news about disasters, and similar topics ( Wutun et al., 2023). ( Sánchez Laws, 2020). Empathetic journalism is journalism that empathizes with people’s suffering, whether caused by culture, social structures, or individuals without boundaries constructed by ethnicity, religion, gender, class, etc. ( Haryanto, 2016) ( Siregar, 2002).

This empathetic journalism is expected to be implemented by media workers and mass media organizations in Kupang City to report on HIV and AIDS cases that position PLWHA as human subjects and not merely statistical objects. Therefore, researchers chose Pos-kupang.com which appears to have a concern with a higher frequency of news coverage on HIV and AIDS compared to other mass media in the city. This frequency of news coverage will be examined whether it is directly proportional to the news content that applies empathetic journalism in it. Sadly, only Pos-kupang.com paid attention to the issue of HIV and AIDS with 43 news reports during 2023, while another cyber media, namely the Antara NTT News Agency ( https://kupang.antaranews.com/) only reported on the topic of HIV and AIDS twice in 2023. Another cyber media, https://www.victorynews.id/, only reported four times during 2023. And the cyber media Timor Express Kupang ( https://timexkupang.fajar.co.id/) also only reported on HIV and AIDS twice in 2023.

The application of empathy journalism by Pos-kupang.com, which pays more attention to the topic of HIV and AIDS compared to other cyber media in Kupang City (NTT), will be analyzed using the framing analysis model of Zhongdang Pan and Gerald M. Kosicki. Pan & Kosicki’s framing mandates that media news be analyzed using four structural frames, namely syntax, script, thematic, and rhetorical ( Fauziati, 2021). HIV and AIDS news broadcast by Pos-kupang.com on World AIDS Day, December 1, 2023, and throughout December 2023, which is identical to World AIDS Month, will be analyzed using the Pan & Kosicki news frame to find the appearance or representation of empathy journalism that is seen in the way journalists compile facts, the way journalists tell facts, the way journalists write facts, and the way journalists emphasize facts.

## Methods

This study uses the framing analysis research method of Zhongdan Pan and Gerald M. Kosicki who operationalizes four structural dimensions of news text as framing devices, namely Syntactic, Script, Thematic and Rhetorical Dimensions ( Siagian & Ritonga, 2024). These four structural dimensions form a kind of theme that links the semantic elements of the news narrative in a global coherence ( Eriyanto & Konstruksi, 2002). This model assumes that each news item has a frame that functions as a center for organizing ideas. A frame is an idea that is connected to different elements in the news text - source quotes, background information, the use of certain words or sentences - into the text as a whole ( Sobur, 2001). Frames are related to meaning. How someone interprets an event can be seen from the set of signs that appear in the text.

The focus of this research is the news framing carried out by cyber media according to the Pan & Kosicki framing device so that the representation of empathy journalism in HIV and AIDS news broadcast by Pos-kupang.com cyber medicine on World AIDS Day, December 1, 2023, can be described. This representation is also elaborated or analyzed using the Media Content Influence Theory from Pamela J. Shoemaker and Stephen D. Reese so that the media’s position in producing news by applying empathy journalism can be revealed.

### Types of research

This research is built on a qualitative research method defined as research that uses a natural setting, with the aim of interpreting the phenomena that occur and is carried out by involving various existing methods ( Creswell, 2019). Qualitative research is conducted by utilizing open-ended interviews to examine and understand the attitudes, views, feelings and behavior of individuals or groups of people ( Moleong, 2014).

### Data collection procedures

The research data collection technique consists of virtual observation and documentation ( Ekaputra, 2022) of Pos-Kupang.com mass media coverage on World HIV/AIDS Day, December 1st. Furthermore, this study also collected news related to HIV/AIDS broadcast during December 2023. These two data collection techniques were used because they are in line with the principles of the research method used, namely Pan & Kosicki’s framing analysis.

### Data analysis

The data analysis techniques used in this study follow the opinion expressed by John W Creswell (2019), namely 1) Processing and preparing data for analysis (data obtained from the research field); 2) Reading all data; 3) Writing coding of all data. 4) Applying the coding process to describe the setting (domain, participants, categories and themes to be analyzed), 5) Showing a descriptive method to connect between themes that will be presented again in a qualitative narrative/report; 6) Interpretation of themes in the research report ( Creswell, 2019). The data analysis steps by Cresswell are elaborated with data analysis techniques according to the mandate of the research method, namely Pan and Kosicki’s framing analysis which consists of analysis of syntactic structure, script structure, thematic structure and rhetorical structure ( Munif, 2023).

### Research object

The research object or unit of analysis in this research proposal is HIV and AIDS news broadcast by Pos-Kupang.com cyber media which can be accessed at https://kupang.tribunnews.com/during December 2023. The reason for choosing December as the unit of analysis is because December is synonymous with the commemoration of World AIDS Day. Therefore, this research attempts to find and reveal the reality of media bias towards HIV and AIDS issues through its reporting. Based on the results of observations on Pos-Kupang.com news during December, only eight news items were found that broadcast about HIV/AIDS cases. The eight news items are presented in table 1 below.

**Table 1.** HIV and AIDS News on Pos-kupang.com December 2023.

Edition	News title	URL/Access Link
Friday, December 1, 2023 4:34 PM WIB	HIV/AIDS Case in Lembata: Activists Request Grant Funds to be Managed by KPAD	https://kupang.tribunnews.com/2023/12/01/kasus-hivaids-di-lembata-pegiat-minta-dana-hibah-untuk-dikelola-kpad
Friday, December 1, 2023 5:32 PM WIB	Commemorating World AIDS Day, PLHIV in Belu Asked Not to Be Discriminated	https://kupang.tribunnews.com/2023/12/01/peringatan-hari-aids-sedunia-odha-di-belu-minta-jangan-didiscriminasi
Saturday, December 2, 2023 10:58 PM WIB	OMK Mayor B Region Invites Ernesto Aldo to Discuss HIV/AIDS at Tablolong	https://kupang.tribunnews.com/2023/12/02/omk-wilayah-walikota-b-ajak-ernesto-aldo-diskusi-hivaids-di-tablolong
Saturday, December 2, 2023 1:51 PM WIB	The number of HIV/AIDS sufferers in Alor Regency reaches 664 people.	https://kupang.tribunnews.com/2023/12/02/penderita-hivaids-di-kabupaten-alor-sentuh-angka-664-orang
Tuesday, December 5, 2023 07:10 WIB	Teenagers’ promiscuity in Lembata is no longer normal.	https://kupang.tribunnews.com/2023/12/05/free-social-among-youth-in-lembata-is-not-reasonable
Tuesday, December 5, 2023 12:48 WIB	16HAKtP Campaign: Kupang Women’s Prison Inmates Educated on TPKS and HIV/AIDS	https://kupang.tribunnews.com/2023/12/05/kampanye-16haktp-warga-binaan-lapas-perempuan-kupang-diedukasi-tpks-dan-hivaids
Tuesday, December 5, 2023 1:56 PM WIB	Shocking! 500 Teenagers Become Prostitutes in Lembata, NTT. What’s Going On?	https://kupang.tribunnews.com/2023/12/05/mencengangkan-lebih-500-remaja-jadi-prostitutes-di-lembata-ntt-ada-apa
Saturday, December 9, 2023, 5:00 PM WIB	Bethesda Yakkum CD, along with the Silawan Village Government and WPA, distributes basic food aid to people living with HIV.	https://kupang.tribunnews.com/2023/12/09/cd-bethesda-yakkum-bersama-pemdes-silawan-dan-wpa-salurkan-bantuan-sembako-untuk-odhiv

*Source:* Pos Kupang.com, 2023

Based on the virtual observations conducted throughout December 2023 ( table 1), a total of eight news articles specifically addressing HIV/AIDS issues were identified on the online media platform Pos-Kupang.com. The distribution of these publications initiated precisely on World AIDS Day, December 1, 2023, with two stories focusing on the dynamics of case management in Lembata Regency and an anti-discrimination plea for PLHIV in Belu Regency. Following this, on December 2, 2023, the coverage shifted to a youth community discussion in Tablolong and a statistical report revealing that the number of sufferers had reached 664 in Alor Regency. The reporting intensity peaked on December 5, 2023, with three simultaneous articles highlighting sensitive and sensational topics, including adolescent promiscuity, TPKS legal education for female prison inmates, and a shocking report regarding teenage prostitution in Lembata. This sequence of publications concluded on December 9, 2023, with a story covering a humanitarian aid distribution of basic food supplies to PLHIV by Bethesda Yakkum and the local village government. All active URLs for these eight news items were systematically documented as the primary units of analysis to facilitate data verification and text framing processes.

### Ethical considerations and human subjects statement

No human participants, field interviews, clinical trials, or focus group discussions were conducted by the researchers. All analyzed data originates from public open-access texts published online by Pos-Kupang.com. Consequently, institutional review board (IRB) ethical approval and participant informed consent protocols were not applicable to this study design. No private personal data or individual profiles were utilized.

## Results

The results of this study are presented in four areas of study consisting of an analysis of the representation of empathy journalism in the way journalists compile facts, tell facts, write facts and emphasize facts based on the Pan & Kosicki framing perspective.

### Representation of empathetic journalism in HIV and AIDS News on Pos-kupang.com based on how journalists compile facts

The results of this study describe the representation of empathetic journalism from the way journalists compile facts so that the aspects that will be explored are the syntactic structure/dimensions in the Pan and Kosicki framing framework which includes headlines, leads, background information, quotes, sources, statements and the closing section of the news.

### Headline

Journalists’ choice of diction in writing news headlines is key to capturing reader interest. The presentation of facts through compelling headlines serves as a key entry point for readers to think and decide to read the story, believing it’s worth reading. This is evident in every headline used in the news published by poskupang.com. In News 1, the title used emphasizes the handling of HIV/AIDS cases in Lembata as a force to attract the reader’s interest to then decide to open the news.

Furthermore, in the headline of news 2, the compilation of facts is directed at the existence of a movement specifically to commemorate World AIDS Day in Belu, which is always held. In this headline, the journalist also wrote to voice the request of PLWHA that there be no discrimination from society against them. If observed carefully, the headline of news 2 can be seen as a form of application of empathetic journalism. This is in line with the perspective of Ashadi Siregar (2002) who stated that empathetic journalism is not only used by journalists in reporting about people with HIV/AIDS alone. Empathetic journalism is journalism that empathizes with people’s suffering, whether caused by culture, social structure, or individual without boundaries constructed by ethnicity, religion, gender, class, and others. Headline of news 2 is a reflection of empathetic journalism where in the headline, the journalist compiles facts and writes the news from the perspective of PLWHA who ask not to be discriminated against in their social environment.

In Headline 4, this news is quite interesting because the journalist includes the number of HIV/AIDS sufferers in Alor Regency. Through this headline, the journalist also wants to demonstrate that public health issues, particularly those related to infectious diseases such as HIV/AIDS, are a major concern in Alor Regency. This high number of sufferers indicates a very serious challenge that must be addressed immediately, because if not addressed quickly, it will continue to increase and have a greater impact. The headline and content of news item 4 are structured to provide information about HIV/AIDS prevention campaign activities. In news 5, the news headline was composed by focusing on the case of free association among teenagers in Lembata.

In the headline of news item 6, the journalist indicates that there is an active effort to disseminate information or conduct campaign actions related to the goal of providing education about HIV/AIDS. Educating indicates that the inmates received education or information on two main topics: TPKS (Criminal Acts of Sexual Violence) and HIV/AIDS. This indicates an effort to increase understanding and awareness of sexual violence and HIV/AIDS-related health issues among female inmates at Kupang Prison. When examined from the perspective of empathy journalism that seeks to provide encouragement, build optimism in life, and support, this news does not prioritize the element of empathy purely because the news was compiled only focusing on campaign activities carried out by PKBI NTT, the UDN foundation and the Women’s House at Kupang Women’s Prison, without providing information on the distribution of PLWHA as found in several poskupang.com news items that highlight information on the distribution of PLWHA.

In Headline News 7, the journalist tried to describe a shocking event that occurred in Lembata, East Nusa Tenggara (NTT), which involved 500 teenagers involved in prostitution practices. This news headline focuses on the promiscuity of teenagers in Lembata. Judging from several other articles published on poskupang.com, this story is more or less similar to the one written by journalist Eflin Rote, which describes the conditions of promiscuity among teenagers in Lembata. This news is framed by highlighting the number of teenagers suspected of working as street sex workers. This can be seen as an empathetic and concerned journalist’s effort to observe teenagers’ social behavior to prevent them from falling into the dangers of HIV/AIDS. Furthermore, in headline 8, the journalist provides information about who is involved (Bethesda Yakkum CD, Silawan Village Government, and WPA), what they are doing (distributing basic food aid), and who the aid is intended for (PLHIV). This provides an overview of the humanitarian activities carried out by various parties to help people affected by HIV/AIDS in their social environment.

### Lead

The lead used in news 1 consists of two, namely the where lead and the when lead. The use of the where lead in News 1 is motivated by the journalist’s desire to emphasize the location of the spread of HIV/AIDS, namely in Lembata Regency with the largest number of people living with HIV/AIDS in Nubatukan District. News 1 also uses the when lead to explain the time span of the development and spread of HIV/AIDS until 2023, which amounted to 183 PLWHA with the largest number of 90 people in Nubatukan District. Furthermore, the when lead is also found in the journalist’s writing which explains the number of deaths of 174 sufferers caused by HIV/AIDS in the period 2008–2023.

In news 2, using 3 lead models namely what lead, where lead and when lead. The use of these 3 leads wants to emphasize that at the beginning of the news sentence it wants to convey that the commemoration of World HIV/AIDS Day was carried out simply and the activity was covered by the journalist in Belu Regency on December 1st which is commemorated as World HIV/AIDS Day. This news lead also wants to highlight what are the desires and hopes of HIV/AIDS sufferers to all groups and there is no more discrimination they get from the environment, so that together they can fight this pandemic in Belu Regency through real action and hard work.

News 3 also uses three forms of lead, namely the what lead, where lead, and when lead to explain that there is a collaboration between the Catholic OMK and the One Frequency Community by showing a collaborative spirit and showing the importance of discussion in the context of public health such as HIV/AIDS. Furthermore, news 4 uses the where lead, what lead, and who lead. In this news 4 lead, the news sentence used by the journalist is to rewrite about HIV/AIDS in Alor Regency which is the same as the news headline. This wants to show that the author wants to emphasize that the number of HIV/AIDS sufferers in Alor Regency is not a small number and there must be seriousness from all parties to solve this problem.

Furthermore, in news item 5, the news lead used is a where lead that explains Lembata as an area with a concerning level of promiscuity, and a what lead that explains the challenges in preventing HIV and AIDS in Lembata. The news lead covers the core issues discussed in the entire news, namely adolescent promiscuity, exposure to pornography and free sex, and their impact on HIV/AIDS prevention. This provides a brief overview to readers about the focus and urgency of the news, which can also be seen as a form of empathy from journalists in compiling the news. In news item 6, the leads used are a where lead and what lead to convey information that at the Kupang Women’s Prison, inmates are receiving socialization and education about the TPKS Law and HIV/AIDS.

In news article 7, the journalist uses the “what” and “where” leads, designed to grab the reader’s attention with a bombastic opening. The word “Astonishing!” in the lead emphasizes the surprise or unusual nature of the event. This is a common strategy used in headlines to immediately grab the reader’s attention. The “where” lead then appears in the sentence “more than 500 teenagers in Lembata Regency, East Nusa Tenggara (NTT) Province.” This statement provides information about where the event occurred, displaying the specific number “more than 500” to suggest that this issue is an important one to address. In news article 8, the “when” lead is used, indicating that this activity was held to commemorate World AIDS Day, which falls on December 1st. Furthermore, the “where” and “who” leads are used, explaining the parties involved, namely the Silawan Village Government and the AIDS Care Community, mentioned in the news as the organizers of the activity. Upon further investigation, it can be explained that this news was structured to provide facts about the Silawan Village Government’s joint activities with PLHIV.

### Statement

In news 2, statements from two sources were used, namely Yosafa as Coordinator of CD Yakkum Bethesda Belu and Budi, a person living with HIV/AIDS. Through this statement, I would like to inform you that in Belu Regency, there was a simple commemoration for World HIV/AIDS Day and through this commemoration, there was a statement conveying that in that year, sufferers expressed great hope and an invitation to work together to fight the pandemic in Belu Regency.

In news 4, there are 3 sources who provide statements including Dominggus Prakameng SKM (Secretary of the Alor Regency KPAD), dr. Farida as the Head of the Alor Regency Health Office and Otto Nodi Atyanto as the General Manager of the Alor Regency Bethesda CD. From this statement it can be concluded that the number of HIV/AIDS sufferers in Alor Regency has increased significantly every year. This can be seen that since 2000–2023 sufferers have reached 664 people. Furthermore, news 6 uses a statement from Septerhani who in his speech appreciated the PKBI NTT which has collaborated with the Kupang Women’s Prison to provide socialization and education for correctional inmates. While in news 8 uses a quote from Dr. Aloysius Haleserens’ statement who is the deputy regent of Belu regarding the target for handling HIV/AIDS in the future where the source is confident that before 2030 the HIV/AIDS problem in Belu Regency can be resolved.

### Closing

In the closing section, news 1 displays a statement from Paskalis Witak regarding the process of HIV/AIDS transmission and an explanation of HIV/AIDS. News 1 states that HIV/AIDS is not transmitted through the air or ordinary physical contact but the transmission process is at least through three mediums, namely blood, genital fluids (male and female) and breast milk (ASI). Furthermore, the closing section in news 2 is written by showing Budi’s proactive attitude in facing the negative stigma against PLWHA in Belu, where he invites the community to open up and eliminate their stigma against HIV/AIDS sufferers or so-called PLWHA. In the closing sentence of news 3, it is composed by conveying the results of records from the NTT Central Statistics Agency regarding the increasingly alarming increase in HIV/AIDS sufferers. It was recorded that in 2017, as many as 245 HIV/AIDS sufferers came from the city of Kupang with a total of 894 PLWHA. In the closing section of news 4, it is composed by highlighting the campaign route starting from St. Yoseph Kalabahi Catholic High School to Kalabahi Mini Field and how the enthusiasm of the participants who took part in the campaign.

In news 5, the closing section ends with a statement about how these teenage sex workers work and even form their own sex worker group. By presenting information about the group and the coordinator, the conclusion encourages the reader to think about further actions that can be taken to address the problem. This could trigger a more effective social response or government action to address the issues faced by teenage street sex workers. Furthermore, in News 6, the closing section provides a brief summary of the event’s events. Participants received material on two important topics: Law Number 12 of 2022 concerning Criminal Acts of Sexual Violence (TPKS), presented by Wati Bagang from the Kupang Women’s House Foundation, and material on HIV/AIDS.

Furthermore, in the closing section of news 7, the complexity of health issues related to HIV/AIDS in Lembata is highlighted. This expression provides a brief understanding of how HIV works in the body, namely by reducing the effectiveness of antibodies and causing susceptibility to various diseases. Meanwhile, in news 8, the closing section of the news ends with an explanation indicating that the problem of HIV and AIDS is still a serious concern in Belu Regency, with an increase in cases from year to year. These data provide an indication that prevention, early detection, and treatment efforts continue to be an important focus in addressing HIV/AIDS issues.

### Representation of empathetic journalism in HIV and AIDS News on Pos-Kupang.com Based on how journalists emphasize facts

In the analysis conducted by researchers on eight news items broadcast by pos-kupang.com, the representation of empathetic journalism can be seen through the journalists’ emphasis on facts in each news item written. Therefore, to examine the emphasis on facts in news on pos-kupang.com, the dimension analyzed is **the rhetorical dimension** with the analysis unit consisting of **lexicon** (words) and **idioms** related to HIV/AIDS. Thus, the researcher did not discuss matters unrelated to HIV/AIDS so that the discussion could focus on the display of empathetic journalism representation in the broadcast news. Of the eight broadcast news stories, there was the use of the same abbreviations and terms in emphasizing facts, namely the words HIV and AIDS, which consist of several syllables and form a single sentence. The word ‘HIV’ is an abbreviation for *Human Immunodeficiency Virus*, meaning a virus that attacks the immune system, which can weaken the body’s ability to fight infections and diseases, which then ultimately will cause the body’s immunity to be impaired. Meanwhile, the word ‘AIDS’ is an abbreviation for *Acquired Immune Deficiency Syndrome*, meaning a condition where the HIV virus has reached the final stage of infection or a syndrome or collection of symptoms caused by a weakened immune system ( Rozak, 2022).

In addition to the use of the terms HIV and AIDS, the eight news reports generally also used the word stigma in each news item to describe situations where people living with HIV/AIDS face rejection in society. In several news reports, stigma is then explained as the cause of people living with HIV/AIDS feeling discriminated against in their social environment, as stigma means prejudice that discredits or rejects a person or group because it is considered different from the general public. The use of the word stigma appears in news 3 which can be seen as a form of emphasis on facts by journalists in writing news which is also a representation of empathetic journalism namely through the use of the sentence ‘stay away from the disease and its behavior, not stay away from the person’. Another term that also appears in news 5 is ‘free association’ where free association is defined as a form of deviant behavior by referring to the term “free” which means going beyond the boundaries of existing norms. The use of this term is done by journalists to emphasize facts in an effort to provide education related to HIV/AIDS prevention. The next term that is also used by journalists in emphasizing facts appears in the term Commercial Sex Workers (CSW) which when seen in news 7 refers to teenagers who do sex work on the streets or commercially ( Chaidir & Tuapattinaja, 2018). The meaning of this term is an individual who sells his sexual services in public places such as streets. This can be interpreted as meaning that in each region, only a small number of people living with HIV/AIDS are known to have HIV/AIDS because other people living with HIV/AIDS are not recorded and are reluctant to seek medical attention at health facilities ( Mukarromah & Azinar, 2021). Furthermore, in news stories 2 and 7, the journalists emphasized the facts through the use of metaphors such as “fighting the HIV/AIDS pandemic”. The metaphor of war is used to describe the efforts or hard work in overcoming HIV/AIDS by considering it as an enemy that must be fought. In this context, this metaphor reflects the colossal and organized effort required to control and stop the spread of HIV/AIDS, much like a war effort undertaken to defeat a threatening enemy.

## Discussion

### Interpretation of main findings

The practice of empathy journalism that appears in Pos-Kupang.com news through the way journalists compile news, write news, narrate news and emphasize news when analyzed using the Theory of Media Content Influence Hierarchy as in the perspective of Pamela J. Shoemaker and Stephen D. Reese (1996) (Shoemaker & Stephen, nd), then there are two main categories that influence the content of Pos-Kupang.com news, namely the influence of individual media workers and the influence of media routines ( Krisdinanto, 2014). The findings of this study indicate that the content/content of news compiled, written and broadcast on pos-kupang.com about HIV/AIDS during December 2023 was influenced by two categories/levels of influence, namely the influence of media workers as individuals and the influence of media routines. At the level of individual influence as media workers, it can be explained that journalists (media workers) as individuals in compiling and writing news cannot be separated from the subjectivity of the journalists themselves ( Rusadi, 2013). Subjectivity in writing HIV/AIDS news is related to knowledge, perspective, experience and certain moral standards possessed by journalists so that it greatly influences the content of the news that is compiled and written ( Mulyana, 2002). Thus, if the element of subjectivity embedded in the individual journalist as a media worker will greatly determine the extent of the level of concern for PLWHA which will later have an impact on the application of empathy journalism in the news produced. Therefore, in relation to news writing by pos-kupang.com journalists, it can be explained that the influence of the individual level which includes the characteristics of communication workers, professional background and personality, as well as personal attitudes and professional roles of journalists has a significant impact on the news produced by journalists, especially in writing news that is empathetic to PLWHA.

Furthermore, at the level of media routine, it can be seen through the news of pos-kupang.com which can be categorized as a routine ( Ratuloli et al., 2023) to present news about HIV/AIDS, especially in December. With this routine, it is clear that pos-kupang.com media will provide a special column containing HIV/AIDS news every year. Therefore, media routines that are not followed by social sensitivity by media workers will not produce news written from the perspective of People Living with HIV/AIDS (PLWHA). At the level of media routine ( Latif & Sos, 2022), research findings show that journalists in producing HIV/AIDS news are also influenced by daily communication practices including *deadlines* (time limits), space requirements in publication, the inverted pyramid structure for writing news, news values, standards of objectivity and journalists’ trust in news sources. In terms of news values, mass media reporting should present news that can reduce stigma and discrimination against PLWHA in society by highlighting the struggles of their lives. Thus, through reporting that prioritizes humanistic values from the perspective of PLWHA, the media has demonstrated its role and concern for public health through the practice of health journalism as reflected in its journalistic works.

### Comparison with previous research

The findings of this study are consistent with the findings of Isibor & Ajuwon (2004) who showed the fact that journalists in Ibadan do not have adequate knowledge about AIDS, and many of them show negative attitudes towards PLWHA, thus weakening their potential ability to educate the public about AIDS. Advocacy workshops and training are recommended to address this problem ( Isibor & Ajuwon, 2004). In this study, when viewed from the news produced, it can be explained that Pos-Kupang journalists are considered to have low sensitivity towards HIV/AIDS and PLWHA so that this is reflected in the news that prioritizes case statistics and the presence of bombastic titles that actually cause stigma from the community towards people with HIV/AIDS or what is often called People With HIV/AIDS (PLWHA). Therefore, training and instilling empathy values are needed to foster the practice of empathetic journalism in reporting.

### Research limitations

The researchers acknowledge that several limitations need to be acknowledged. First, this research only focuses on the framing of news by journalists so it does not explicitly look at the influence of individual journalists’ subjectivity in producing journalistic work. Second, this study does not examine how media routines influence the news produced by journalists. Third, this research has not specifically examined the motives behind news writing by journalists.

### Recommendations for future research

Further research should focus on the influence of media routines on news content, the influence on news content when viewed from influences outside the media organization, the influence of media ideology on journalists in producing journalistic works and a study of how journalists view empathetic journalism which prioritizes humanist elements through journalistic works.

## Conclusion

The findings of this study indicate that the content of news compiled, written and broadcast on pos-kupang.com about HIV/AIDS during December 2023 was influenced by two categories/levels of influence, namely the influence of media workers as individuals and the influence of media routines. At the level of influence of the individual level, the characteristics of journalists, professional backgrounds and personalities as well as personal attitudes and professional roles of journalists have a significant impact on the news produced by journalists, especially in writing news that empathizes with PLWHA, although there are some journalists who do not show empathy through news that places HIV/AIDS cases in terms of numbers statistically. Meanwhile, at the level of media routines, pos-kupang.com reporting is categorized as a routine that will provide a special column to publish HIV/AIDS news every year without also considering the application of values that reflect the practice of empathetic journalism.

Although this study provides valuable insights into the practice of empathetic journalism in responding to public health issues, several limitations of the study such as the study of the influence of media routines on news content, the influence on news content when viewed from influences outside the media organization, the influence of media ideology on journalists in producing journalistic works and the study of how journalists view empathetic journalism that prioritizes humanist elements through journalistic works can be used as a focus for further research.

## Data Availability

The qualitative text framing matrices, structural coding sheets, and word-frequency tracking datasets supporting the analytical conclusions of this study are openly accessible in the Zenodo public data repository under the https://doi.org/10.5281/zenodo.21753859. ( **masamah, et al., **2026 **).** Dataset Title: Dataset of Article. Data is shared under the Creative Commons Attribution 4.0 International (CC BY 4.0) license.
