# Framing HIV/AIDS: Evaluating Empathetic Journalism in Local Online Media

**DOI:** 10.12688/f1000research.187439.2

**Published:** 2026-10-28

**Authors:** Mas'amah Mas'amah, Monika Wutun, Herman Elfridus Seran, Apris A. Adu, Emanuel Sowe Leuape

**Affiliations:** 1 Communication Science, University of Nusa Cendana, Kupang, East Nusa Tenggara, Indonesia; 2 Communication Science, University of Nusa Cendana, Kupang, East Nusa Tenggara, Indonesia; 3 Communication Science, University of Nusa Cendana, Kupang, East Nusa Tenggara, Indonesia; 4 Public Health Science, University of Nusa Cendana, Kupang, East Nusa Tenggara, Indonesia; 5 Communication Science, University of Nusa Cendana, Kupang, East Nusa Tenggara, Indonesia

**Keywords:** HIV/AIDS, Health Journalism, Mass Media, Empathy, Framing Pan & Kosicki

## Abstract

This study explores the practices of health journalism in mass media coverage of Human Immunodeficiency Virus and Acquired Immunodeficiency Syndrome (HIV/AIDS) during World HIV/AIDS Day on the online media platform Pos-Kupang.com. It evaluates whether online news is written from a perspective of empathy towards ODHA or simply centers on tracking case statistics and reporting negative case distributions. Methods The research employed Zhongdan Pan and Gerald M. Kosicki’s Framing Analysis model, operationalizing four structural text dimensions as framing devices: Syntactic, Script, Thematic, and Rhetorical. Data collection was conducted virtually by selecting all active news stories related to HIV/AIDS published by Pos-Kupang.com during December 2023. The framing analysis revealed that only two out of eight news stories integrated empathetic journalism, which was operationally identified by headlines written from the PLHIV perspective, active counter-stigmatization narratives, and the positioning of PLHIV as human subjects rather than passive victims. The remaining six articles failed to meet these criteria, instead employing sensationalized framing (such as focusing on teenage prostitution) and heavy reliance on quantitative case numbers. The rhetorical devices frequently relied on war metaphors and stigmatizing lexicons. This framing pattern was directly influenced by individual journalist subjectivity—specifically a lack of health communication training—and rigid media routines driven by strict deadlines and the traditional inverted-pyramid structure. While local online media consistently allocate coverage for World AIDS Month, they largely fail to embed empathetic journalism values due to structural limitations. The reporting heavily commodifies human suffering into statistical objects and sensational headlines. Consequently, this study fills a theoretical gap by expanding Shoemaker and Reese’s hierarchy of influence into local health journalism. Practically, it provides an evidence-based recommendation for online editorial boards to move away from quantitative-centric framing and implement specialized health journalism training to foster non-discriminatory, supportive public health communication.

## Introduction

The increase in HIV and AIDS cases is a worrying phenomenon. This is evidenced by the increasing number of cases each year. It is important to note that humans have lived side by side with HIV and AIDS since it was first discovered globally in 1920 and in Indonesia in 1987 ( Andina, 2019). Data on the development of the increase in cases in Indonesia has increased significantly from year to year. At the time of this study, data from the Indonesian Ministry of Health (Kemenkes) recorded that the number of HIV cases in Indonesia was projected to reach 515,455 cases during January–September 2023 ( Hasibuan et al., 2024). Of this total, 454,723 cases, or 88%, had been confirmed by ODHA or people living with HIV (PLHIV). The Ministry of Health stated that only 40% of people living with HIV (PLHIV) are receiving HIV treatment. By age, the majority of ODHA in Indonesia are in the 25–49 age group, accounting for 69.9% of the total cases. The second largest age group is the 20–24 age group, at 16.1%. This is followed by those aged 50 and over at 7.7% and those aged 15–19 at 3.4%. Children, including toddlers or those aged 4 and over, account for 1.9% and those aged 5–14 at 1% ( Pratiwi et al., 2022). The alarming rise of HIV/AIDS cases is highly visible in Kupang City, the provincial capital of East Nusa Tenggara (NTT). In public health management, mass media serves as a strategic channel to disseminate preventive education and eliminate social biases ( Solihin et al., 2023).

Ideally, cyber media utilizes its rapid outreach to shape supportive public opinions toward marginalized health subjects ( Indriani & Zainuddin, 2025). However, a significant gap exists between ideal health communication and local journalistic realities. Local cyber media in NTT rarely report on HIV/AIDS. When coverage emerges, it heavily favors quantitative data and bombastic angles over the actual lived experiences of the ODHA ( Yani et al., 2020). Preliminary virtual observations in 2023 revealed that major local outlets such as Antara NTT, Victory News, and Timor Express published fewer than four HIV/AIDS articles annually. In contrast, Pos-kupang.com demonstrated a higher commitment by publishing 43 articles throughout 2023. Nevertheless, high coverage frequency does not guarantee empathetic depth. Prior studies on local journalism have largely ignored how media framing reduces human suffering into statistical metrics during critical advocacy periods like World AIDS Month. This lack of empirical evaluation on empathetic health reporting constitutes the primary research gap of this study.

The reality obtained by researchers even on World AIDS Day on December 1st each year, there are only a few media that pay attention by broadcasting news about HIV and AIDS. And from the results of these observations, it was found that local mass media categorized as cyber media that paid attention to the issue of HIV and AIDS by broadcasting this news twice on World AIDS Day was Pos-kupang.com which can be accessed at https://kupang.tribunnews.com/. Meanwhile, other cyber mass media in Kupang City only reported once or did not report topics related to HIV and AIDS at all. The search was carried out again by researchers to find news about HIV and AIDS in mass media in NTT which have editorial headquarters in Kupang City and it turned out the harsh reality was found. Not all media treat HIV and AIDS as a common problem. In a period of one year (12 months) alone, the frequency of news on the same topic by mass media in the province and Kupang City was very limited.

This limited number of news items raises the question of whether all news published by the mass media in Kupang City is “friendly” to HIV and AIDS. Empathetic journalism operates as an alternative reportorial paradigm that rejects traditional detached objectivity when covering deeply traumatized or marginalized human subjects. In health crisis reporting, empathy in journalism is not mere emotional sympathy; rather, it is a deliberate cognitive and ethical commitment by media workers to understand and project the structural suffering of vulnerable groups without socio-cultural boundaries ( Haryanto, 2016; Siregar, 2002). According to Sánchez Laws, 2020, this framework requires journalists to actively dismantle the psychological distance between the reader and the subject, thereby triggering a supportive civic response. Empathetic reporting is structurally operationalized through three critical dimensions: first, the prioritization of ‘people-first narratives’ that view patients as active human subjects rather than mechanical epidemiological tallies; second, the intentional integration of counter-stigmatization language; and third, the balanced representation of humanitarian struggles ( Wutun et al., 2023). In local online ecosystems, implementing this approach serves as a critical counter-weight to routine, click-driven sensationalism.

This empathetic journalism is expected to be implemented by media workers and mass media organizations in Kupang City to report on HIV and AIDS cases that position PLWHA as human subjects and not merely statistical objects. Therefore, researchers chose Pos-kupang.com which appears to have a concern with a higher frequency of news coverage on HIV and AIDS compared to other mass media in the city. This frequency of news coverage will be examined whether it is directly proportional to the news content that applies empathetic journalism in it.

Therefore, this study aims to examine the structural representation of empathetic journalism in HIV/AIDS news reports published by Pos-kupang.com during the World AIDS Month cycle in December 2023. Utilizing Zhongdan Pan and Gerald M. Kosicki’s qualitative framing matrix, this study disassembles news texts across four structural operational layers: syntactic arrangements, script formulations, thematic progressions, and rhetorical emphasis ( Fauziati, 2021). This investigation offers a dual contribution. Theoretically, it expands the boundaries of Shoemaker and Reese’s hierarchy of media influence theory by explicitly testing it within the under-researched ecosystem of local digital health reporting in eastern Indonesia. Practically, the resulting evidence provides empirical recommendations for local online editorial boards to design non-stigmatizing, supportive, and human-centric health communication frameworks.

## Methods

This study uses the framing analysis research method of Zhongdan Pan and Gerald M. Kosicki who operationalizes four structural dimensions of news text as framing devices, namely Syntactic, Script, Thematic and Rhetorical Dimensions ( Siagian & Ritonga, 2024). These four structural dimensions form a kind of theme that links the semantic elements of the news narrative in a global coherence ( Eriyanto & Konstruksi, 2002). This model assumes that each news item has a frame that functions as a center for organizing ideas. A frame is an idea that is connected to different elements in the news text - source quotes, background information, the use of certain words or sentences - into the text as a whole ( Sobur, 2001). Frames are related to meaning. How someone interprets an event can be seen from the set of signs that appear in the text.

To fully comprehend why empathetic values succeed or fail to manifest in news texts, this study employs Pamela J. Shoemaker and Stephen D. Reese’s Hierarchy of Influence Theory as an analytical bridge. Media content is never produced in a vacuum; it is shaped by five nested layers of influence: individual media workers, media routines, organizational policies, extra-media forces, and macro-ideological systems ( Shoemaker & Reese, 1996). This study specifically focuses on the interplay between the individual level (the journalists’ personal empathy and health communication training) and the media routines level (news formats, strict deadlines, and the inverted-pyramid structure). The structural integration between Shoemaker and Reese's framework and Zhongdan Pan and Gerald M. Kosicki’s framing model lies in a cause-and-effect relationship. Pan and Kosicki’s framing devices (syntactic, script, thematic, and rhetorical dimensions) function as the textual mirror reflecting internal organizational dynamics. In other words, media routines and individual subjectivity act as the internal determinants (the causes), while the resulting text frames—whether they reduce PLHIV into statistical objects or elevate them through empathetic narratives—serve as the empirical outputs (the effects). By synthesized these two models, this study can systematically trace how rigid corporate routines in local newsrooms directly dictate the structural choices of words, headlines, and thematic structures in HIV/AIDS advocacy reporting.

### Types of research

This study adopts a qualitative descriptive approach utilizing Zhongdan Pan and Gerald M. Kosicki’s text framing analysis method ( Fauziati, 2021). The unit of analysis is defined strictly as the individual online news articles published by the cyber media platform Pos-Kupang.com. To capture the full spectrum of high-stakes health advocacy reporting, the temporal boundary of the dataset was set during the World AIDS Month cycle from December 1 to December 31, 2023. The text is treated as an empirical manifestation of newsroom dynamics, which is systematically deconstructed to evaluate the textual representation of empathetic journalism frameworks.

### Data collection procedures

Data collection was conducted using virtual observation and comprehensive digital archiving methods. Sample selection was executed via a purposive sampling strategy based on four strict inclusion criteria: (1) the article must be published on the active domain of Pos-Kupang.com; (2) the publication date must fall within December 2023; (3) the headline or body text must explicitly target HIV/AIDS issues; and (4) the coverage must be localized within the East Nusa Tenggara (NTT) administrative perimeter. A digital keyword search using 'HIV', 'AIDS', 'ODHA', and 'ODHIV' was run on the internal search engine of Pos-Kupang.com. Out of 43 general health publications monitored, a final sample of eight news stories strictly met all inclusion criteria and was documented for structural framing mapping. 

### Data analysis

Data analysis was executed by integrating John W. Creswell's qualitative text steps with the specific structural devices of Pan and Kosicki's framing matrix Creswell (2019). To ensure transparency, trackability, and rigorous peer evaluation, text deconstruction was operationalized using a predefined coding sheet across four structural text layers ( Munif, 2023):

1) Syntactic Structure (Fact Compilation): Analyzed through headlines, leads, background context, and source selection. Empathetic journalism is coded if headlines adopt the PLHIV perspective and utilize active human sources instead of institutional-only voices.
2) Script Structure (Fact Narration): Analyzed using the 5W+1H narrative layout. Empathetic journalism is coded if the narrative flow builds life optimism and highlights human agency rather than passive victimization.
3) Thematic Structure (Fact Writing): Analyzed through paragraph coherence, causal propositions, and secondary data linkage. Empathetic journalism is coded if the text provides structural reasons for the illness rather than moralistic personal judgments.
4) Rhetorical Structure (Fact Emphasis): Analyzed through choice of lexicons, metaphors, and idioms. Empathetic journalism is coded if the text utilizes 'people-first language' (e.g., PLHIV/ODHA) and actively avoids discriminatory terms.

### Research object

The research object or unit of analysis in this research proposal is HIV and AIDS news broadcast by Pos-Kupang.com cyber media which can be accessed at https://kupang.tribunnews.com/during December 2023. The reason for choosing December as the unit of analysis is because December is synonymous with the commemoration of World AIDS Day. Therefore, this research attempts to find and reveal the reality of media bias towards HIV and AIDS issues through its reporting. Based on the results of observations on Pos-Kupang.com news during December, only eight news items were found that broadcast about HIV/AIDS cases. The eight news items are presented in Table 1 below.

**Table 1.** HIV and AIDS News on Pos-kupang.com December 2023.

Edition	News title	URL/Access Link
Friday, December 1, 2023 4:34 PM WIB	HIV/AIDS Case in Lembata: Activists Request Grant Funds to be Managed by KPAD	https://kupang.tribunnews.com/2023/12/01/kasus-hivaids-di-lembata-pegiat-minta-dana-hibah-untuk-dikelola-kpad
Friday, December 1, 2023 5:32 PM WIB	Commemorating World AIDS Day, PLHIV in Belu Asked Not to Be Discriminated	https://kupang.tribunnews.com/2023/12/01/peringatan-hari-aids-sedunia-odha-di-belu-minta-jangan-didiscriminasi
Saturday, December 2, 2023 10:58 PM WIB	OMK Mayor B Region Invites Ernesto Aldo to Discuss HIV/AIDS at Tablolong	https://kupang.tribunnews.com/2023/12/02/omk-wilayah-walikota-b-ajak-ernesto-aldo-diskusi-hivaids-di-tablolong
Saturday, December 2, 2023 1:51 PM WIB	The number of HIV/AIDS ODHA in Alor Regency reaches 664 people.	https://kupang.tribunnews.com/2023/12/02/penderita-hivaids-di-kabupaten-alor-sentuh-angka-664-orang
Tuesday, December 5, 2023 07:10 WIB	Teenagers’ promiscuity in Lembata is no longer normal.	https://kupang.tribunnews.com/2023/12/05/free-social-among-youth-in-lembata-is-not-reasonable
Tuesday, December 5, 2023 12:48 WIB	16HAKtP Campaign: Kupang Women’s Prison Inmates Educated on TPKS and HIV/AIDS	https://kupang.tribunnews.com/2023/12/05/kampanye-16haktp-warga-binaan-lapas-perempuan-kupang-diedukasi-tpks-dan-hivaids
Tuesday, December 5, 2023 1:56 PM WIB	Shocking! 500 Teenagers Become Prostitutes in Lembata, NTT. What’s Going On?	https://kupang.tribunnews.com/2023/12/05/mencengangkan-lebih-500-remaja-jadi-prostitutes-di-lembata-ntt-ada-apa
Saturday, December 9, 2023, 5:00 PM WIB	Bethesda Yakkum CD, along with the Silawan Village Government and WPA, distributes basic food aid to people living with HIV.	https://kupang.tribunnews.com/2023/12/09/cd-bethesda-yakkum-bersama-pemdes-silawan-dan-wpa-salurkan-bantuan-sembako-untuk-odhiv

*Source:* Pos Kupang.com, 2023

Based on the virtual observations conducted throughout December 2023 ( Table 1), a total of eight news articles specifically addressing HIV/AIDS issues were identified on the online media platform Pos-Kupang.com. The distribution of these publications initiated precisely on World AIDS Day, December 1, 2023, with two stories focusing on the dynamics of case management in Lembata Regency and an anti-discrimination plea for PLHIV in Belu Regency. Following this, on December 2, 2023, the coverage shifted to a youth community discussion in Tablolong and a statistical report revealing that the number of ODHA had reached 664 in Alor Regency. The reporting intensity peaked on December 5, 2023, with three simultaneous articles highlighting sensitive and sensational topics, including adolescent promiscuity, TPKS legal education for female prison inmates, and a shocking report regarding teenage prostitution in Lembata. This sequence of publications concluded on December 9, 2023, with a story covering a humanitarian aid distribution of basic food supplies to PLHIV by Bethesda Yakkum and the local village government. All active URLs for these eight news items were systematically documented as the primary units of analysis to facilitate data verification and text framing processes.

### Ethical considerations and human subjects statement

No human participants, field interviews, clinical trials, or focus group discussions were conducted by the researchers. All analyzed data originates from public open-access texts published online by Pos-Kupang.com. Consequently, institutional review board (IRB) ethical approval and participant informed consent protocols were not applicable to this study design. No private personal data or individual profiles were utilized.

## Results

The results of this study are presented in four areas of study consisting of an analysis of the representation of empathy journalism in the way journalists compile facts, tell facts, write facts and emphasize facts based on the Pan & Kosicki framing perspective.

### Representation of empathetic journalism in HIV and AIDS News on Pos-kupang.com based on how journalists compile facts

The results of this study describe the representation of empathetic journalism from the way journalists compile facts so that the aspects that will be explored are the syntactic structure/dimensions in the Pan and Kosicki framing framework which includes headlines, leads, background information, quotes, sources, statements and the closing section of the news.

### Headline

Journalists’ choice of diction in writing news headlines is key to capturing reader interest. The presentation of facts through compelling headlines serves as a key entry point for readers to think and decide to read the story, believing it’s worth reading. This is evident in every headline used in the news published by poskupang.com. In News 1, the title used emphasizes the handling of HIV/AIDS cases in Lembata as a force to attract the reader’s interest to then decide to open the news.

Furthermore, in the headline of news 2, the compilation of facts is directed at the existence of a movement specifically to commemorate World AIDS Day in Belu, which is always held. In this headline, the journalist also wrote to voice the request of PLWHA that there be no discrimination from society against them. If observed carefully, the headline of news 2 can be seen as a form of application of empathetic journalism. This is in line with the perspective of Ashadi Siregar (2002) who stated that empathetic journalism is not only used by journalists in reporting about people with HIV/AIDS alone. Empathetic journalism is journalism that empathizes with people’s suffering, whether caused by culture, social structure, or individual without boundaries constructed by ethnicity, religion, gender, class, and others. The syntactic structure of News 2 explicitly reflects the empirical criteria of empathetic journalism through its headline construction. The text reads: *‘World AIDS Day Commemoration: People Living with HIV/AIDS in Belu Ask Not to Be Discriminated Against’.* Textually, the researcher observes that the headline directly prioritizes the active voice and agency of PLHIV rather than framing them as passive, nameless medical objects. The chosen diction, ‘Ask Not to Be Discriminated Against’, serves as a strong textual anchor that shifts the focus of the news from institutional routines toward a human-centered appeal against social exclusion. Thus, the text itself establishes an empathetic frame by centering the narrative on the subaltern perspective. In Headline 4, this news is quite interesting because the journalist includes the number of HIV/AIDS in Alor Regency. Through this headline, the journalist also wants to demonstrate that public health issues, particularly those related to infectious diseases such as HIV/AIDS, are a major concern in Alor Regency. This high number of ODHA indicates a very serious challenge that must be addressed immediately, because if not addressed quickly, it will continue to increase and have a greater impact. The headline and content of news item 4 are structured to provide information about HIV/AIDS prevention campaign activities. In news 5, the news headline was composed by focusing on the case of free association among teenagers in Lembata.

In the headline of news item 6, the journalist indicates that there is an active effort to disseminate information or conduct campaign actions related to the goal of providing education about HIV/AIDS. Educating indicates that the inmates received education or information on two main topics: TPKS (Criminal Acts of Sexual Violence) and HIV/AIDS. This indicates an effort to increase understanding and awareness of sexual violence and HIV/AIDS-related health issues among female inmates at Kupang Prison. A structural analysis of News 6, titled *‘16HAKtP Campaign: Kupang Women’s Prison Inmates Educated on TPKS and HIV/AIDS*’, reveals a dominant administrative-routine frame rather than an empathetic-humanist frame. Textually, the narrative flow focuses strictly on institutional stakeholders, as evidenced by the source list which only quotes structural figures such as Septerhani from the prison management. The researcher interprets that this journalistic choice produces a ‘communication decoupling’ effect: the text documents the advocacy event but completely silences the voices and lived experiences of the female inmates themselves. Because the text lacks any direct quotes or lived narratives from the vulnerable subjects inside the prison, it fails to meet the operational definition of empathetic journalism, reducing a critical health issue into a routine public relations report.

In contrast, the syntactic device in News 7 employs a sensationalized, quantitative-driven framing structure. The headline states: *‘Shocking! Over 500 Teenagers Become Prostitutes in Lembata, NTT. What’s Going On?’.* Textual observation shows that the use of the exclamation ‘Shocking’ acts as a rhetorical tool designed to induce public anxiety rather than foster public health empathy. While the text purports to address the socio-behavioral environment of youth to prevent HIV transmission, the researcher’s interpretation notes a significant counter-empathetic outcome: the narrative structure heavily commodifies human suffering by reducing complex structural vulnerability into a shocking statistic (Over 500 Teenagers). By linking youth promiscuity directly to an alarmist question (What’s Going On?), the text builds a moralistic frame that distances the reader from the subject, potentially intensifying social stigma against the group under study. Furthermore, in headline 8, the journalist provides information about who is involved (Bethesda Yakkum CD, Silawan Village Government, and WPA), what they are doing (distributing basic food aid), and who the aid is intended for (PLHIV). This provides an overview of the humanitarian activities carried out by various parties to help people affected by HIV/AIDS in their social environment.

### Lead

The lead used in news 1 consists of two, namely the where lead and the when lead. The use of the where lead in News 1 is motivated by the journalist’s desire to emphasize the location of the spread of HIV/AIDS, namely in Lembata Regency with the largest number of people living with HIV/AIDS in Nubatukan District. News 1 also uses the when lead to explain the time span of the development and spread of HIV/AIDS until 2023, which amounted to 183 PLWHA with the largest number of 90 people in Nubatukan District. Furthermore, the when lead is also found in the journalist’s writing which explains the number of deaths of 174 ODHA caused by HIV/AIDS in the period 2008–2023.

In news 2, using 3 lead models namely what lead, where lead and when lead. The use of these 3 leads wants to emphasize that at the beginning of the news sentence it wants to convey that the commemoration of World HIV/AIDS Day was carried out simply and the activity was covered by the journalist in Belu Regency on December 1st which is commemorated as World HIV/AIDS Day. This news lead also wants to highlight what are the desires and hopes of ODHA to all groups and there is no more discrimination they get from the environment, so that together they can fight this pandemic in Belu Regency through real action and hard work.

News 3 also uses three forms of lead, namely the what lead, where lead, and when lead to explain that there is a collaboration between the Catholic OMK and the One Frequency Community by showing a collaborative spirit and showing the importance of discussion in the context of public health such as HIV/AIDS. Furthermore, news 4 uses the where lead, what lead, and who lead. In this news 4 lead, the news sentence used by the journalist is to rewrite about HIV/AIDS in Alor Regency which is the same as the news headline. This wants to show that the author wants to emphasize that the number of HIV/AIDS in Alor Regency is not a small number and there must be seriousness from all parties to solve this problem.

Furthermore, in news item 5, the news lead used is a where lead that explains Lembata as an area with a concerning level of promiscuity, and a what lead that explains the challenges in preventing HIV and AIDS in Lembata. The news lead covers the core issues discussed in the entire news, namely adolescent promiscuity, exposure to pornography and free sex, and their impact on HIV/AIDS prevention. This provides a brief overview to readers about the focus and urgency of the news, which can also be seen as a form of empathy from journalists in compiling the news. In news item 6, the leads used are a where lead and what lead to convey information that at the Kupang Women’s Prison, inmates are receiving socialization and education about the TPKS Law and HIV/AIDS.

In news article 7, the journalist uses the “what” and “where” leads, designed to grab the reader’s attention with a bombastic opening. The word “Astonishing!” in the lead emphasizes the surprise or unusual nature of the event. This is a common strategy used in headlines to immediately grab the reader’s attention. The “where” lead then appears in the sentence “more than 500 teenagers in Lembata Regency, East Nusa Tenggara (NTT) Province.” This statement provides information about where the event occurred, displaying the specific number “more than 500” to suggest that this issue is an important one to address. In news article 8, the “when” lead is used, indicating that this activity was held to commemorate World AIDS Day, which falls on December 1st. Furthermore, the “where” and “who” leads are used, explaining the parties involved, namely the Silawan Village Government and the AIDS Care Community, mentioned in the news as the organizers of the activity. Upon further investigation, it can be explained that this news was structured to provide facts about the Silawan Village Government’s joint activities with PLHIV.

### Statement

In news 2, statements from two sources were used, namely Yosafa as Coordinator of CD Yakkum Bethesda Belu and Budi, a person living with HIV/AIDS. Through this statement, I would like to inform you that in Belu Regency, there was a simple commemoration for World HIV/AIDS Day and through this commemoration, there was a statement conveying that in that year, ODHA expressed great hope and an invitation to work together to fight the pandemic in Belu Regency.

In news 4, there are 3 sources who provide statements including Dominggus Prakameng SKM (Secretary of the Alor Regency KPAD), dr. Farida as the Head of the Alor Regency Health Office and Otto Nodi Atyanto as the General Manager of the Alor Regency Bethesda CD. From this statement it can be concluded that the number of ODHA in Alor Regency has increased significantly every year. This can be seen that since 2000–2023 ODHA have reached 664 people. Furthermore, news 6 uses a statement from Septerhani who in his speech appreciated the PKBI NTT which has collaborated with the Kupang Women’s Prison to provide socialization and education for correctional inmates. While in news 8 uses a quote from Dr. Aloysius Haleserens’ statement who is the deputy regent of Belu regarding the target for handling HIV/AIDS in the future where the source is confident that before 2030 the HIV/AIDS problem in Belu Regency can be resolved.

### Closing

In the closing section, news 1 displays a statement from Paskalis Witak regarding the process of HIV/AIDS transmission and an explanation of HIV/AIDS. News 1 states that HIV/AIDS is not transmitted through the air or ordinary physical contact but the transmission process is at least through three mediums, namely blood, genital fluids (male and female) and breast milk (ASI). Furthermore, the closing section in news 2 is written by showing Budi’s proactive attitude in facing the negative stigma against PLWHA in Belu, where he invites the community to open up and eliminate their stigma against ODHA or so-called PLWHA. In the closing sentence of news 3, it is composed by conveying the results of records from the NTT Central Statistics Agency regarding the increasingly alarming increase in ODHA. It was recorded that in 2017, as many as 245 ODHA came from the city of Kupang with a total of 894 PLWHA. In the closing section of news 4, it is composed by highlighting the campaign route starting from St. Yoseph Kalabahi Catholic High School to Kalabahi Mini Field and how the enthusiasm of the participants who took part in the campaign.

In news 5, the closing section ends with a statement about how these teenage sex workers work and even form their own sex worker group. By presenting information about the group and the coordinator, the conclusion encourages the reader to think about further actions that can be taken to address the problem. This could trigger a more effective social response or government action to address the issues faced by teenage street sex workers. Furthermore, in News 6, the closing section provides a brief summary of the event’s events. Participants received material on two important topics: Law Number 12 of 2022 concerning Criminal Acts of Sexual Violence (TPKS), presented by Wati Bagang from the Kupang Women’s House Foundation, and material on HIV/AIDS.

Furthermore, in the closing section of news 7, the complexity of health issues related to HIV/AIDS in Lembata is highlighted. This expression provides a brief understanding of how HIV works in the body, namely by reducing the effectiveness of antibodies and causing susceptibility to various diseases. Meanwhile, in news 8, the closing section of the news ends with an explanation indicating that the problem of HIV and AIDS is still a serious concern in Belu Regency, with an increase in cases from year to year. These data provide an indication that prevention, early detection, and treatment efforts continue to be an important focus in addressing HIV/AIDS issues.

### Representation of empathetic journalism in HIV and AIDS News on Pos-Kupang.com Based on how journalists emphasize facts

In the analysis conducted by researchers on eight news items broadcast by pos-kupang.com, the representation of empathetic journalism can be seen through the journalists’ emphasis on facts in each news item written. Therefore, to examine the emphasis on facts in news on pos-kupang.com, the dimension analyzed is **the rhetorical dimension** with the analysis unit consisting of **lexicon** (words) and **idioms** related to HIV/AIDS. Thus, the researcher did not discuss matters unrelated to HIV/AIDS so that the discussion could focus on the display of empathetic journalism representation in the broadcast news. Of the eight broadcast news stories, there was the use of the same abbreviations and terms in emphasizing facts, namely the words HIV and AIDS, which consist of several syllables and form a single sentence. The word ‘HIV’ is an abbreviation for *Human Immunodeficiency Virus*, meaning a virus that attacks the immune system, which can weaken the body’s ability to fight infections and diseases, which then ultimately will cause the body’s immunity to be impaired. Meanwhile, the word ‘AIDS’ is an abbreviation for *Acquired Immune Deficiency Syndrome*, meaning a condition where the HIV virus has reached the final stage of infection or a syndrome or collection of symptoms caused by a weakened immune system ( Rozak, 2022).

In addition to the use of the terms HIV and AIDS, the eight news reports generally also used the word stigma in each news item to describe situations where people living with HIV/AIDS face rejection in society. In several news reports, stigma is then explained as the cause of people living with HIV/AIDS feeling discriminated against in their social environment, as stigma means prejudice that discredits or rejects a person or group because it is considered different from the general public. The use of the word stigma appears in news 3 which can be seen as a form of emphasis on facts by journalists in writing news which is also a representation of empathetic journalism namely through the use of the sentence ‘stay away from the disease and its behavior, not stay away from the person’. Another term that also appears in news 5 is ‘free association’ where free association is defined as a form of deviant behavior by referring to the term “free” which means going beyond the boundaries of existing norms. The use of this term is done by journalists to emphasize facts in an effort to provide education related to HIV/AIDS prevention. The next term that is also used by journalists in emphasizing facts appears in the term Commercial Sex Workers (CSW) which when seen in news 7 refers to teenagers who do sex work on the streets or commercially ( Chaidir & Tuapattinaja, 2018). The meaning of this term is an individual who sells his sexual services in public places such as streets. This can be interpreted as meaning that in each region, only a small number of people living with HIV/AIDS are known to have HIV/AIDS because other people living with HIV/AIDS are not recorded and are reluctant to seek medical attention at health facilities ( Mukarromah & Azinar, 2021). Furthermore, in news stories 2 and 7, the journalists emphasized the facts through the use of metaphors such as “fighting the HIV/AIDS pandemic”. The metaphor of war is used to describe the efforts or hard work in overcoming HIV/AIDS by considering it as an enemy that must be fought. In this context, this metaphor reflects the colossal and organized effort required to control and stop the spread of HIV/AIDS, much like a war effort undertaken to defeat a threatening enemy.

## Discussion

### Interpretation of main findings

The practice of empathy journalism that appears in Pos-Kupang.com news through the way journalists compile news, write news, narrate news and emphasize news when analyzed using the Theory of Media Content Influence Hierarchy as in the perspective of Pamela J. Shoemaker and Stephen D. Reese (1996) (Shoemaker & Stephen, nd), then there are two main categories that influence the content of Pos-Kupang.com news, namely the influence of individual media workers and the influence of media routines ( Krisdinanto, 2014). The findings of this study indicate that the content/content of news compiled, written and broadcast on pos-kupang.com about HIV/AIDS during December 2023 was influenced by two categories/levels of influence, namely the influence of media workers as individuals and the influence of media routines. At the level of individual influence as media workers, it can be explained that journalists (media workers) as individuals in compiling and writing news cannot be separated from the subjectivity of the journalists themselves ( Rusadi, 2013). Subjectivity in writing HIV/AIDS news is related to knowledge, perspective, experience and certain moral standards possessed by journalists so that it greatly influences the content of the news that is compiled and written ( Mulyana, 2002). The empirical distribution of the findings where two out of eight articles successfully integrated empathetic elements while the remaining six did not demonstrates that the expression of empathy is not entirely absent, but rather highly fragmented within local newsrooms. This variance confirms that individual journalist subjectivity is a critical, yet unstable, determinant in health reporting. When journalists possess specific health communication awareness, they successfully bypass routine institutional data to produce human-centered narratives, as seen in the anti-discrimination frames of News 2 and News 8. Conversely, when this individual sensitivity is lacking, journalists default to mechanical media routines, resulting in the structural reduction of PLHIV into statistical objects observed in the other six articles. Therefore, individual professional backgrounds and personal attitudes do not uniformly limit empathy. Instead, they create an internal tension against rigid daily newsroom routines.

Furthermore, at the level of media routine, it can be seen through the news of pos-kupang.com which can be categorized as a routine ( Ratuloli et al., 2023) to present news about HIV/AIDS, especially in December. With this routine, it is clear that pos-kupang.com media will provide a special column containing HIV/AIDS news every year. Therefore, media routines that are not followed by social sensitivity by media workers will not produce news written from the perspective of People Living with HIV/AIDS (PLWHA). At the level of media routine ( Latif & Sos, 2022), research findings show that journalists in producing HIV/AIDS news are also influenced by daily communication practices including *deadlines* (time limits), space requirements in publication, the inverted pyramid structure for writing news, news values, standards of objectivity and journalists’ trust in news sources. In terms of news values, mass media reporting should present news that can reduce stigma and discrimination against PLWHA in society by highlighting the struggles of their lives. Thus, through reporting that prioritizes humanistic values from the perspective of PLWHA, the media has demonstrated its role and concern for public health through the practice of health journalism as reflected in its journalistic works.

### Comparison with previous research

The findings of this study are consistent with the findings of Isibor & Ajuwon (2004) who showed the fact that journalists in Ibadan do not have adequate knowledge about AIDS, and many of them show negative attitudes towards PLWHA, thus weakening their potential ability to educate the public about AIDS. Advocacy workshops and training are recommended to address this problem Rather than indicating a uniform 'low sensitivity' across all media workers, these findings illustrate a state of communication decoupling driven by organizational constraints. The presence of two empathetic articles proves that local journalists hold the latent capacity for humanistic reporting. However, this capacity is frequently overshadowed by structural levels of influence, such as tight deadlines and space requirements, which force journalists to prioritize pre-packaged case statistics and institutional press releases over time-consuming field interviews with PLHIV. This alignment underscores that the issue is less about an inherent individual apathy among local journalists and more about the lack of structured health journalism training within the newsroom framework to systematically sustain empathetic reporting practices. Therefore, training and instilling empathy values are needed to foster the practice of empathetic journalism in reporting. Therefore, training and instilling empathy values are needed to foster the practice of empathetic journalism in reporting.

### Research limitations

Several limitations in this study’s design must be explicitly acknowledged to contextualize the findings. First, because this research relies strictly on a purposive sample of eight news articles published by a single local platform during a one-month cycle, the findings cannot be generalized to represent the broader attitudes or empathy levels of all journalists in Kupang City or Indonesia. Second, while the text framing mirrors the internal determinants of reporting, this study did not conduct direct field interviews or ethnography to measure journalists’ internal psychological motives or the exact corporate pressures of the newsroom. Consequently, the scope of this argument is tightly bounded to the textual outputs of the framing matrix during World AIDS Month, and further empirical research is mandatory to test these institutional variables across wider temporal and organizational scales.

### Recommendations for future research

Further research should focus on the influence of media routines on news content, the influence on news content when viewed from influences outside the media organization, the influence of media ideology on journalists in producing journalistic works and a study of how journalists view empathetic journalism which prioritizes humanist elements through journalistic works.

## Conclusion

This study concludes that while local online mass media channels successfully allocate routine reporting spaces for annual health advocacy cycles like World AIDS Month, they largely fail to embed empathetic journalism values into the text. The analytical framing model by Pan and Kosicki systematically proves that media texts heavily commodify individual human suffering into mechanical case statistics and alarmist, stigmatizing headlines. By decoding these textual outputs through Shoemaker and Reese's hierarchy of influence theory, this research reveals a structural dominance where rigid daily media routines (such as strict deadlines, publishing spaces, and traditional inverted-pyramid formats) routinely overshadow the latent empathetic capacities of individual journalists.

The conceptual contribution of this study is twofold. Theoretically, it fills an empirical gap by expanding the application of the hierarchy of media influence framework into the context of localized cyber health journalism in eastern Indonesia, demonstrating that high coverage volume does not automatically equate to humanistic depth. Practically, these findings carry direct implications for modern journalistic practices, proving that standard detached objectivity in local newsrooms accidentally perpetuates social biases and public distrust toward marginalized groups like PLHIV.

Based on these empirical findings, this study offers three actionable, evidence-based recommendations: (1) local online editorial boards must immediately implement specialized health communication and empathetic journalism training for field reporters; (2) newsroom routines must transition away from quantitative-centric and sensationalized headings toward 'people-first narratives' that amplify patient agency; and (3) regional public health institutions must actively collaborate with cyber media gates to supply non-discriminatory, structurally-grounded information kits rather than raw numerical tallies.

## Data Availability

The qualitative text framing matrices, structural coding sheets, and word-frequency tracking datasets supporting the analytical conclusions of this study are openly accessible in the Zenodo public data repository under the https://doi.org/10.5281/zenodo.21753859 ( masamah, et al., 2026). Dataset Title: Dataset of Article. Data is shared under the Creative Commons Attribution 4.0 International (CC BY 4.0) license.
