# Supplementary material for: Framing HIV/AIDS: Evaluating Empathetic Journalism in Local Online Media
Source: F1000Res. 2026 Oct 28;15:1329. Originally published 2026 Aug 7. [Version 2] doi: 10.12688/f1000research.187439.2 (PMC13691354; doi:10.12688/f1000research.187439.2)
Supplement: Peer review history

# Reviewer report: Reviewer response for version 2

**DOI:** 10.5256/f1000research.213257.r534686

**Published:** 2026-10-30

**Authors:** Junaidi Awang Besar

**Affiliations:** 1 Universiti Kebangsaan Malaysia, Bangi, Selangor, Malaysia

All proposed improvements have been implemented well and are complete.

Is the work clearly and accurately presented and does it cite the current literature?

Yes

If applicable, is the statistical analysis and its interpretation appropriate?

Yes

Are all the source data underlying the results available to ensure full reproducibility?

Partly

Is the study design appropriate and is the work technically sound?

Yes

Are the conclusions drawn adequately supported by the results?

Partly

Are sufficient details of methods and analysis provided to allow replication by others?

Yes

Reviewer Expertise:

Social Geography

I confirm that I have read this submission and believe that I have an appropriate level of expertise to confirm that it is of an acceptable scientific standard.

# Reviewer report: Reviewer response for version 1

**DOI:** 10.5256/f1000research.206873.r524304

**Published:** 2026-10-22

**Authors:** Novel Lyndon

**Affiliations:** 1 Universiti Kebangsaan Malaysia, Bangi, Selangor, Malaysia

**Article Title:** HIV/AIDS, Media, and Health Journalism: Exploring Empathetic Journalism Practices in HIV/AIDS Reporting on the Mass Media

**Summary of the Article and Its Relevance**

 This study analyzes empathetic journalism across eight HIV/AIDS news reports published by Pos-Kupang.com in December 2023. Using Pan and Kosicki’s (1993) framing model and Shoemaker and Reese’s hierarchy of influences framework, the authors examine how news reports structure information and discuss potential factors shaping coverage.

 The authors conclude that only two articles substantially highlight the struggles of people living with HIV (PLHIV) against stigma, arguing that reporting prioritizes statistics over lived experiences. The study offers valuable insights into local Indonesian health journalism. However, it requires clearer analytical criteria, systematic evidence presentation, and more cautious conclusions.

**Strengths of the Article**

 Addresses an important ethical topic: dignity, agency, and voice in health reporting for PLHIV.

 Uses framing analysis appropriately to evaluate headlines, lead paragraphs, source selection, and tone.

 Provides good transparency in Table 1 with publication details and direct links to analyzed articles.

 Acknowledges key limitations regarding the lack of direct data on newsroom routines.

**Major Comments**

 1. Define and Operationalize "Empathetic Journalism" (pp. 3–4, 6–8)

 State explicit, observable criteria used to classify coverage as showing empathy, limited empathy, or stigmatizing framing (e.g., inclusion of PLHIV perspectives, non-stigmatizing language, privacy protections).

 Map these criteria directly to Pan and Kosicki’s four framing dimensions.

 Avoid assuming that reporting routine statistics or official events automatically indicates a lack of empathy; evaluate how this information is framed.

 2. Clarify Article Selection and Coding Procedures (pp. 4–5)

 Provide specific search terms, dates, and retrieval methods used on Pos-Kupang.com.

 State clear inclusion/exclusion criteria, including how broader topics (e.g., sex work, adolescent health) qualified as HIV-related news.

 Specify the coding unit (e.g., full article, paragraph, quotation) and detail who coded the material, how categories were developed, and how inter-coder reliability or consistency was managed.

 3. Present All Four Framing Dimensions Systematically (pp. 4, 6–8)

 Ensure equal analytical weight across all four dimensions (syntactic, script, thematic, rhetorical). Currently, findings heavily emphasize syntactic and rhetorical features.

 Include a summary matrix mapping all eight articles across the four dimensions to ensure consistent application.

 4. Reconcile Abstract Claims with Results (pp. 1–2, 6–8)

 Explicitly identify the two articles categorized as centering lived experiences and explain the criteria used.

 Move beyond a simple present/absent binary for empathy to discuss mixed cases containing both empathetic and problematic elements.

 5. Evaluate Tone and Language More Critically (pp. 6–8)

 Do not automatically equate an educational or preventive goal with empathy; examine whether language about sexuality or sex work carries moral judgment or blame.

 Separate narrative text written by journalists from direct source quotes, assessing whether quotes are challenged or uncritically reproduced.

 Support interpretations with direct text extracts and explain Indonesian-to-English translations where relevant.

 6. Align Organizational Claims with Study Scope (pp. 2, 8–10)

 Text analysis cannot establish causal newsroom factors or journalists' personal attitudes. Present the hierarchy of influences framework as a possible explanatory lens rather than a confirmed empirical finding.

 Remove or support claims that the outlet maintains a "dedicated annual HIV/AIDS column."

 Adjust claims in the Abstract, Discussion, and Conclusion to match the textual evidence.

 7. Distinguish Textual Potential from External Audience Effects (pp. 3, 9)

 Avoid stating that specific headlines directly cause stigma, as audience reception was not measured. Use qualified language (e.g., "may reinforce existing stigma").

 Frame journalist training recommendations as practical proposals rather than proven interventions.

 8. Connect Repository Data and Restrict Regional Scope (pp. 5–10)

 Explain how the Zenodo repository files (coding sheets, word-frequency data) connect to specific findings.

 Explicitly frame the paper as a single-outlet qualitative case study over one month rather than generalizing to Indonesian media as a whole.

**Minor Comments**

 Title (p. 1): Simplify for conciseness (e.g., "Empathetic Journalism in HIV Reporting: A Framing Analysis of Pos-Kupang.com").

 Citations & History (p. 3): Clarify the reference to HIV being "first discovered in 1920" by distinguishing early 20th-century origin estimates from clinical identification in 1981. Ensure epidemiological statistics distinguish between cumulative cases, active diagnoses, and prevalence.

 Terminology (throughout): Use person-first language (people living with HIV) consistently. Maintain clear distinctions between HIV and AIDS.

 Table 1 (p. 5): Number entries (1–8) to match the text, and verify translated titles, dates, and URLs.

 Consistency (pp. 4–5, 10): Remove references to "interviews" or "informants" in the Methods and Acknowledgements to align with the document-based nature of the study.

 Editing (throughout): Correct repetitive phrasing and non-standard terms (e.g., "cyber medicine"), and verify reference list formatting and theorist citations.

**Overall Assessment**

 This manuscript addresses an important topic in health communication. Its focus on voice, representation, and dignity in local media reporting is a strong contribution. Resolving analytical ambiguities, ensuring methodological transparency, and aligning conclusions strictly with the textual evidence will significantly strengthen the paper for indexing.

Is the work clearly and accurately presented and does it cite the current literature?

Yes

If applicable, is the statistical analysis and its interpretation appropriate?

Yes

Are all the source data underlying the results available to ensure full reproducibility?

Yes

Is the study design appropriate and is the work technically sound?

Yes

Are the conclusions drawn adequately supported by the results?

Yes

Are sufficient details of methods and analysis provided to allow replication by others?

Yes

Reviewer Expertise:

My research focuses on community and rural development, particularly community empowerment, sustainable livelihoods, social capital, indigenous knowledge, and inclusive participation. I examine how development policies and local institutions shape socioeconomic wellbeing, community resilience, and equitable access to opportunities among rural and indigenous communities.

I confirm that I have read this submission and believe that I have an appropriate level of expertise to confirm that it is of an acceptable scientific standard.

# Reviewer report: Reviewer response for version 1

**DOI:** 10.5256/f1000research.206873.r526527

**Published:** 2026-10-19

**Authors:** Junaidi Awang Besar

**Affiliations:** 1 Universiti Kebangsaan Malaysia, Bangi, Selangor, Malaysia

**Aspect**

**Title**

**Identified Issues: **The phrase “Exploring Empathetic Journalism Practices” could be refined to improve its precision and academic tone.

**Recommended Improvements: **Adopt a more concise and focused title.

**Abstract**

**Identified Issues: **The findings and conclusions are relatively general and do not clearly explain the criteria used to identify empathetic journalism.

**Recommended Improvements: **Present the key findings based on the analysis of the eight news articles.

**Introduction**

**Identified Issues: **The section contains repetitive information, overly long sentences, and several claims that require stronger supporting sources.

**Recommended Improvements: **Organise the discussion around the research context, research gap, objectives, and contributions of the study.

**Literature Review**

**Identified Issues: **The concepts of empathetic journalism and the hierarchy of influences on media content are not discussed in sufficient depth.

**Recommended Improvements: **Clarify the conceptual definitions and explain the relationship between the two analytical frameworks.

**Methodology**

**Identified Issues: **The procedures for article selection, units of analysis, coding categories, and analytical reliability are not adequately explained.

**Recommended Improvements: **Describe the analytical process systematically to ensure that the study is transparent, traceable, and open to critical evaluation.

**Findings**

**Identified Issues: **The analysis is largely descriptive and occasionally infers journalists’ intentions without direct supporting evidence.

**Recommended Improvements: **Distinguish textual observations from the researchers’ interpretations and support interpretations with quotations from the news articles.

**Discussion**

**Identified Issues: **There is an apparent inconsistency between the finding that only two news articles highlight the struggles of people living with HIV (PLHIV) and the broader claim that journalists generally demonstrate limited empathy.

**Recommended Improvements: **Ensure that the arguments are consistent with the available evidence and the limitations of the research design.

**Ethics and Terminology**

**Identified Issues: **Terms such as “sufferers,” “HIV/AIDS patients,” and “PLWHA” are used inconsistently and may contribute to stigmatisation.

**Recommended Improvements: **Prioritise the term “people living with HIV (PLHIV)” and adopt non-discriminatory, person-centred language throughout the manuscript.

**Conclusion**

**Identified Issues: **The section reiterates the findings and limitations without adequately explaining the theoretical and practical implications of the study.

**Recommended Improvements: **Summarise the study’s contributions, implications for journalistic practice, and evidence-based recommendations.

Is the work clearly and accurately presented and does it cite the current literature?

Yes

If applicable, is the statistical analysis and its interpretation appropriate?

Yes

Are all the source data underlying the results available to ensure full reproducibility?

Partly

Is the study design appropriate and is the work technically sound?

Yes

Are the conclusions drawn adequately supported by the results?

Partly

Are sufficient details of methods and analysis provided to allow replication by others?

Yes

Reviewer Expertise:

Social Geography

I confirm that I have read this submission and believe that I have an appropriate level of expertise to confirm that it is of an acceptable scientific standard.

# Response

**Published:** 2026-10-20

**Authors:** Mas'amah Mas'amah

**Affiliations:** Communication Science, University of Nusa Cendana, Kupang, East Nusa Tenggara, Indonesia

Dear Reviewer,

 We would like to express our sincere gratitude for your rigorous, constructive, and insightful evaluation of our manuscript. Your feedback has been invaluable in transforming this paper from a descriptive report into a theoretically grounded and methodologically transparent piece of health communication research.

 We have addressed every concern and implemented all substantive revisions in the updated manuscript. Below is a detailed point-by-point response outlining the specific changes made to the substance of the text:

**1. Title and Abstract Realignment**

- **Reviewer Comment:** *The phrase “Exploring Empathetic Journalism Practices” needs enhancement for academic tone. The title should be more concise and focused. The abstract's results and conclusions are too general and lack clear identification criteria.*
- **Response & Revision Substance:** We agree with the critique. We have revised the title to **"Framing HIV/AIDS: Evaluating Empathetic Journalism in Local Online Media"**, which removes redundancies and establishes a rigorous academic focus. In the **Abstract**, we completely replaced the generic summaries with explicit operational criteria for empathetic journalism (e.g., PLHIV-centered headlines, active counter-stigmatization narratives, and human agency). The **Results** section now details the exact breakdown of the 8 articles (2 integrating empathy vs. 6 relying on sensationalism and quantitative metrics), and the **Conclusions** explicitly state the study’s theoretical expansion and actionable practical recommendations.

**2. Introduction and Literature Review Enhancement**

- **Reviewer Comment:** *The introduction contains repetitive information and long sentences; claims lack strong literature support. The concept of empathetic journalism and the hierarchy of influence are not discussed deeply; the relationship between the two frameworks is missing.*
- **Response & Revision Substance:** We have thoroughly restructured the **Introduction** around four clear pillars: research context, empirical gaps, objectives, and dual contributions. We broke down long, compound sentences into active, short, and precise academic declarations, while removing unverified institutional claims. In the **Literature Review**, we replaced superficial definitions with a deep conceptual exploration of empathetic journalism based on three distinct operational dimensions. Crucially, we constructed a robust **theoretical bridge explaining the causal relationship** between the two frameworks: Shoemaker and Reese's Hierarchy of Influence (individual and routine levels) acts as the internal determinant ( *the cause*), while Pan and Kosicki’s framing devices (syntactic, script, thematic, rhetorical) serve as the empirical textual mirror ( *the effect*).

**3. Methodological Transparency and Replicability**

- **Reviewer Comment:** *Procedures for article selection, unit of analysis, coding categories, and analytical reliability are not adequately explained. The analytical process must be systematic and open to critical evaluation.*
- **Response & Revision Substance:** The **Methods** section has been completely rewritten to ensure absolute transparency and full trackability. We have explicitly detailed: (1) a qualitative descriptive research design; (2) a purposive sampling strategy with four strict inclusion criteria; (3) a detailed operational coding sheet specifying what constitutes empathetic vs. non-empathetic traits across the four structural text layers; and (4) a qualitative **inter-coder triangulation consensus protocol** conducted by two independent researchers from the team to eliminate subjective bias and ensure descriptive validity.

**4. Results: Separating Textual Observations from Interpretations**

- **Reviewer Comment:** *Analysis is mostly descriptive and speculates on journalists’ intent without direct supporting evidence. Researcher interpretations must be separated from textual data and supported by direct news quotes.*
- **Response & Revision Substance:** We have completely purged the text of any psychological speculation regarding the "internal intent" of journalists. The **Results** section has been restructured to strictly separate empirical textual observations from researcher interpretations. Every interpretive claim is now anchored directly in **concrete textual evidence and verbatim quotes** from the analyzed news items (e.g., explicitly contrasting the human-centered anchor *'Minta Jangan Didiskriminasi'* in News 2 against the sensationalist, anxiety-inducing exclamation *'Mencengangkan!'* in News 7).

**5. Discussion and Conclusion: Logical Consistency and Contributions**

- **Reviewer Comment:** *There is a clear logical inconsistency between finding only two empathetic articles and claiming broadly that journalists generally show limited empathy. The conclusion merely repeats findings without showing theoretical and practical implications.*
- **Response & Revision Substance:** We have rectified this logical flaw by narrowing the scope of our argument. The **Discussion** no longer makes sweeping over-generalizations about all media workers; instead, it reframes the variance (2 out of 8 articles) as an empirical tension between latent individual empathy and rigid, corporate newsroom routines. We have fully expanded the **Research Limitations** sub-section to acknowledge the boundaries of our small purposive sample size. Finally, the **Conclusion** has been completely rewritten to move away from descriptive repetition. It now explicitly delineates the study's **theoretical contribution** (expanding media influence layers in eastern Indonesia) and **practical implications**, culminating in **three distinct, evidence-based actionable recommendations** for cyber editorial boards and public health gates.

We believe these comprehensive revisions have drastically elevated the substance, rigor, and scholarly value of our manuscript. We hope the updated paper now meets the high standards of the journal.

                                                                                                                                       Sincerely,

**The Authors**
